# Pre-operative fluid resuscitation in the emergency general surgery septic patient: does it really matter?

**DOI:** 10.1186/s12873-021-00479-3

**Published:** 2021-07-22

**Authors:** Benjamin Moran, Erin Major, Joseph A. Kufera, Samuel A. Tisherman, Jose Diaz

**Affiliations:** 1 Einstein Healthcare Network, Sidney Kimmel Medical College at Thomas Jefferson University, Einstein Medical Center, Klein Building, Suite 101, 5401 Old York Road, Philadelphia, PA 19141 USA; 2 R Adams Cowley Shock Trauma Center, University of Maryland School of Medicine, Baltimore, MD USA

**Keywords:** Emergency general surgery, Abdominal Sepsis, Fluid resuscitation, Source control, Pre-operative resuscitation

## Abstract

**Objective:**

Emergency general surgery (EGS) patients presenting with sepsis remain a challenge. The Surviving Sepsis Campaign recommends a 30 mL/kg fluid bolus in these patients, but recent studies suggest an association between large volume crystalloid resuscitation and increased mortality. The optimal amount of pre-operative fluid resuscitation prior to source control in patients with intra-abdominal sepsis is unknown. This study aims to determine if increasing volume of resuscitation prior to surgical source control is associated with worsening outcomes.

**Methods:**

We conducted an 8-year retrospective chart review of EGS patients undergoing surgery for abdominal sepsis within 24 h of admission. Patients in hemorrhagic shock and those with outside hospital index surgeries were excluded. We grouped patients by increasing pre-operative resuscitation volume in 10 ml/kg intervals up to > 70 ml/kg and later grouped them into < 30 ml/kg or ≥ 30 ml/kg. A relative risk regression model compared amounts of fluid administration. Mortality was the primary outcome measure. Secondary outcomes were time to operation, ventilator days, and length of stay (LOS). Groups were compared by quick Sequential Organ Failure Assessment (qSOFA) and SOFA scoring systems.

**Results:**

Of the 301 patients included, the mean age was 55, 51% were male, 257 (85%) survived to discharge. With increasing fluid per kg (< 10 to < 70 ml/kg), there was an increasing mortality per decile, 8.8% versus 31.6% (*p* = 0.004). Patients who received < 30 mL/kg had lower mortality (11.3 vs 21%) than those who received > 30 ml/kg (*p* = 0.02). These groups had median qSOFA scores (1.0 vs. 1.0, *p* = 0.06). There were no differences in time to operation (6.1 vs 4.9 h *p* = 0.11), ventilator days (1 vs 3, *p* = 0.08), or hospital LOS (8 vs 9 days, *p* = 0.57). Relative risk regression correcting for age and physiologic factors showed no significant differences in mortality between the fluid groups.

**Conclusions:**

Greater pre-operative resuscitation volumes were initially associated with significantly higher mortality, despite similar organ failure scores. However, fluid volumes were not associated with mortality following adjustment for other physiologic factors in a regression model. The amount of pre-operative volume resuscitation was not associated with differences in time to operation, ventilator days, ICU or hospital LOS.

## Background

Emergency General Surgery (EGS) patients presenting with urgent need of source control and signs of sepsis remain a clinical challenge. These patients require adequate resuscitation to tolerate the induction of general anesthesia however cannot undergo prolonged resuscitation as they need urgent source control to prevent further deterioration. Despite this being a longstanding problem in surgery, no true optimum amount of pre-operative resuscitation has been defined. There are a variety of recommendations outlining the amount of proper pre-operative resuscitation in this septic patient population however no true guidelines have been established.

The Surviving Sepsis Campaign and the most recent international consensus for sepsis and septic shock (sepsis-3) define sepsis as a life-threatening organ dysfunction caused by a dysregulated host response to infection, with septic shock being a subset of sepsis with circulatory and cellular/metabolic dysfunction [1, 2]. Fluid resuscitation in sepsis changed in 2001, with the implementation of early goal directed therapy, with sequential fluid boluses to achieve a desired central venous pressure and has subsequently evolved throughout the years [3]. In the latest rendition, the Surviving Sepsis Campaign guidelines recommends a treatment bundle in patients with hypotension, elevated lactate, and signs of sepsis which includes at least 30 mL/kg of IV crystalloid, broad spectrum antibiotics and vasopressor use to maintain mean arterial pressure greater than 65 [1, 4, 5]. Conversely, recent reports have shown increasing mortality high fluid administration and positive fluid balances [6–8]. The World Society of Emergency Surgery recommends prompt diagnosis of sepsis, with adequate resuscitation and total amount of initial resuscitation defined by clinical response without specific fluid goals, except for optimizing tissue perfusion [9]. The optimal amount of pre-operative fluid resuscitation prior to source control is unknown. This study aims to determine if a certain volume of resuscitation prior to surgical source control in septic patients is associated with worsening outcomes.

## Methods

A retrospective review was conducted of a prospectively collected acute care surgery registry at the University of Maryland Medical Center and R Adams Cowley Shock Trauma Center from May 2011 to July 2018.

Patients requiring urgent surgery within 24 h of admission for surgical source control of intra-abdominal infection were included and only the index admission was included for each patient. These surgeries included, but were not limited to hollow viscous perforation, intra-abdominal abscess, and strangulated bowel. Patients undergoing surgery for trauma, hemorrhagic shock, or had their index surgery at an outside hospital were excluded. All patients were resuscitated either prior to transport to our facility, at our University Hospital, or experienced a combination of resuscitation at both locations. The total amount of pre-operative crystalloid fluid resuscitation was recorded. Patients who needed blood transfusions or colloid resuscitation were excluded.

The patients were grouped by increasing volume of resuscitation, in 10 mL/kg intervals, up to greater than 70 mL/kg prior to operation. The subjects were later dichotomized into less than or greater than or equal to 30 mL/kg. The electronic medical record (Epic) was reviewed for additional data points. Data collected included presenting vital signs, vasopressor use, height, weight, laboratory values (total bilirubin, creatinine, lactate, platelet count, PaO2) and fractional inspired oxygen requirement. Groups were stratified by quick Sequential Organ Failure Assessment (qSOFA) and standard SOFA scoring systems [10, 11].

Mortality was the primary outcome measure for both groupings. Secondary outcomes were measured in the greater or less than 30 mL/kg groups, including time to operation or source control, ventilator days, intensive care unit length of stay, and total hospital length of stay.

Bivariate analysis was used to determine mortality per increasing 10 ml/kg of fluid with Mantel-Haenszel chi-square analysis to determine significance for the trend. Chi-square and Fisher’s exact tests were used to compare non-ordinal categorical factors between groups. Continuous data were presented in terms of medians and the interquartile range (IQR; 25th -75th percentile) and were compared between groups using the Wilcoxon rank-sum test. Multivariable Poisson regression models were fit to determine the association between fluid level and mortality, with and without adjustment by additional covariates. The relationship of each independent variable with mortality was estimated by the relative risk and its 95% confidence interval.

Permission to conduct this study was obtained from the University of Maryland, Baltimore, Institutional Review Board. As this was a study collected from a previously collected database, the need for written, informed consent was waived by the Institutional Review Board of the University of Maryland. As human subjects were involved, this study protocol was performed in accordance with the relevant guidelines set by the University of Maryland.

## Results

**Fig. 1:**
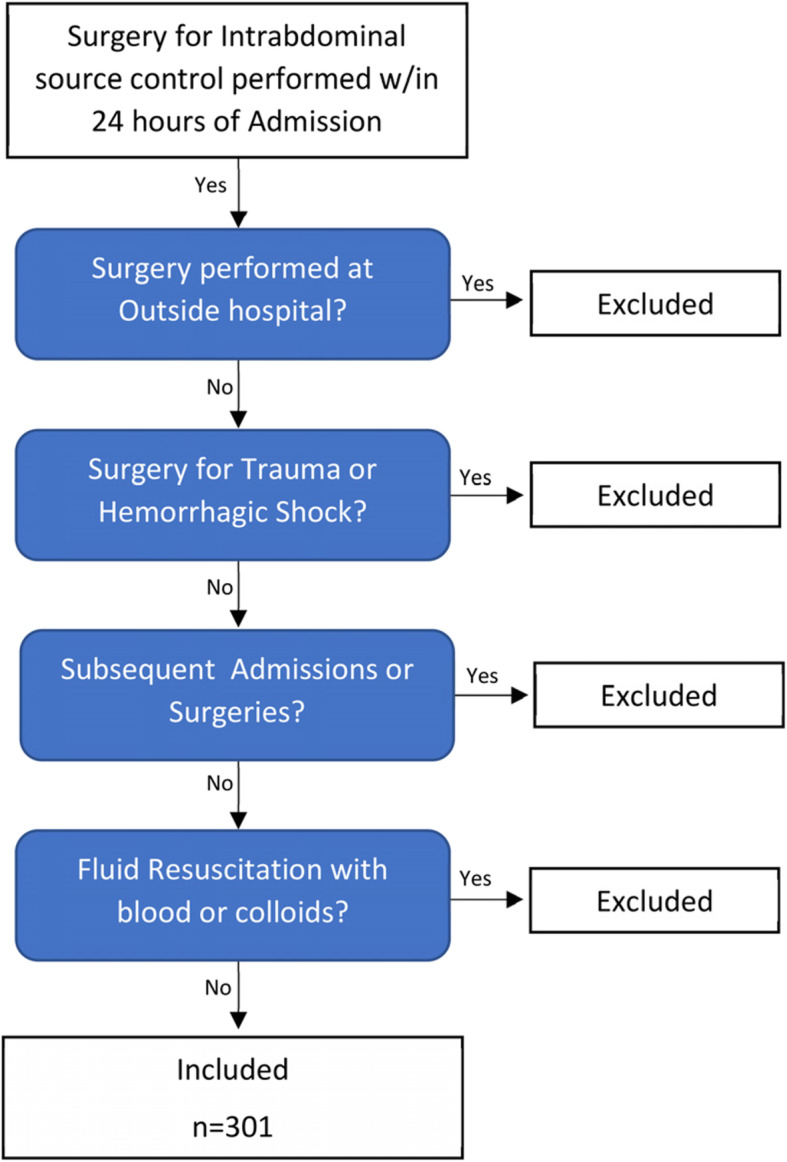
Study Design, Inclusion/Exclusion

In 7 years, 301 patients met our inclusion criteria for the study (Fig. 1, Table 1). Of the patients included, 152 (50.5%) were male, had a median age of 55 (IQR 41–69) years and had a BMI of 29.1 (24.2–36.9) kg/m^2^. Median preoperative vital signs included a mean arterial pressure of 86.5 mmHg (74–98.5), systolic blood pressure of 121 mmHg (106–137), heart rate of 97 beats/min (84–114), respiratory rate of 18 breaths/min (16–23), and a temperature of 98.4 °F (98.1–99.3). Median preoperative lab values included lactate 1.8 mmol/L (1.3–2.9), creatinine 1.1 mg/dL (0.8–1.8), platelet 230 × 10^3^/mcL (158–311), bilirubin 0.9 mg/dL (0.6–1.5), and a PaO2 130 mmHg (95–175). These patients presented with median SOFA scores of 2 (0–8), and qSOFA scores of 1 (0–2).

**Table 1:** Demographics, Vitals, Laboratory Values, Scoring systems, interventions and outcomes

Variable	Median	Lower Quartile	Upper Quartile
Age (yr)	55	41	69
BMI (kg/m^2^)	29.1	24.2	36.9
HR (beats/min)	97	84	114
MAP (mmHg)	86.5	74	98.5
SBP (mmHg)	121	106	137
RR (breath/min)	18	16	23
Temp (°F)	98.4	98.1	99.3
FiO2 (%)	40	40	50
Bili (mg/dL)	0.9	0.6	1.5
Creatinine (mg/dL)	1.1	0.8	1.8
Lactate (mmol/L)	1.8	1.3	2.9
Plt (×10^3^/mcL)	230	158	311
PaO2 (mkmHg)	130	95	175
SOFA	2	0	8
qSOFA	1	0	2
Time to OR (hour)	5.8	3.3	12.6
Fluids/Kg (mL/Kg)	19.0	9.3	35.3
Ventilator Days	2	0	7
ICU LOS (day)	3	0	12
LOS (day)	9	3	20

*BMI* body mass index, *MAP* mean arterial pressure, *SBP* systolic blood pressure, *FiO2* fractional inspired oxygen, *PaO2* partial pressure of oxygen, *SOFA* sequential organ failure assessment score, *qSOFA* quick sequential organ failure assessment score

The cohort received a median pre-operative fluid resuscitation volume of 19.0 ml/kg (9.3–35.3), and 85 (28%) required pre-operative vasopressor use. All required an urgent operation, within 24 h, with a median time to the OR of 5.8 h (3.3–12.6). Post operatively, these patients experienced average ventilator days of 2 (0–7), intensive care length of stay of 3 d (0–12), and total hospital length of stay 9 d (3–20). Overall mortality was 14.6% (44 patients).

**Fig. 2:**
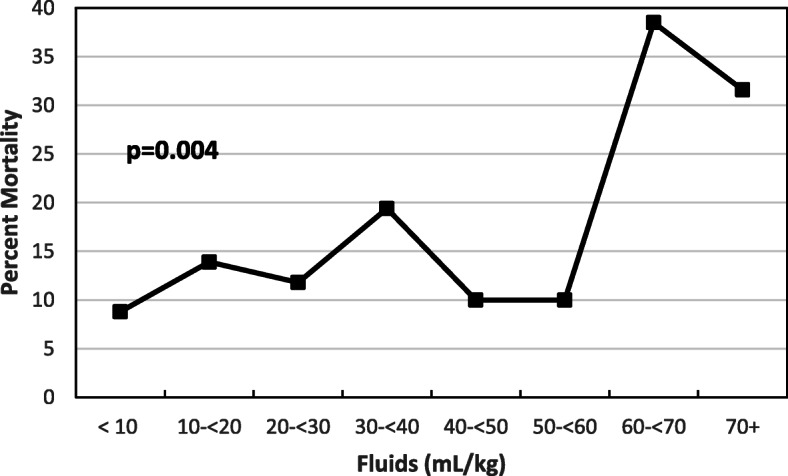
Percent Mortality with increasing 10 ml/kg fluid resuscitation

Patients were grouped based upon pre-operative fluid volume in 10 mL/kg groups, up to greater or equal than 70 mL/kg (Fig. 2). For fluid resuscitation, 80 patients (27%) received less than 10 mL/kg, 72 (24%) received 10- < 20 mL/kg, 51 (17%) received 20- <  30 mL/kg, 36 (12%) received 30- < 40 mL/kg, 20 (7%) received 40- < 50 mL/kg, 10 (3%) received 50- < 60 mL/kg, 13 (4%) received 60- < 70 mL/kg, and 19 (6%) received greater than or equal to 70 mL/kg. Chi-square analysis indicated a significant trend of increasing mortality as fluids per kg increased, *p* = 0.004. Percent mortality and totals in each group is demonstrated by Table 2.

**Table 2:** Percent Mortality with increasing 10 ml/kg fluid resuscitation

Fluids per Kg by Mortality			
**Fluid per Kg**	**Survived**	**Died**	**Total**
**< 10 mL/kg**	73 (91.3%)	7 (8.8%)	80
**10- < 20 mL/kg**	62 (86.1%)	10 (13.9%)	72
**20- < 30 mL/kg**	45 (88.2%)	6 (11.8%)	51
**30- < 40 mL/kg**	29 (80.6%)	7 (19.4%)	36
**40- < 50 mL/kg**	18 (90.0%)	2 (10.0%)	20
**50- < 60 mL/kg**	9 (90.0%)	1 (10.0%)	10
**60- < 70 mL/kg**	8 (61.5%)	5 (38.5%)	13
**70+ mL/kg**	13 (68.4%)	6 (31.6%)	19
**Total**	257	44	301
**Statistic**	DF	Value	Prob
**Mantel-Haenszel Chi-Square**	1	8.4864	0.0036

**Fig. 3:**
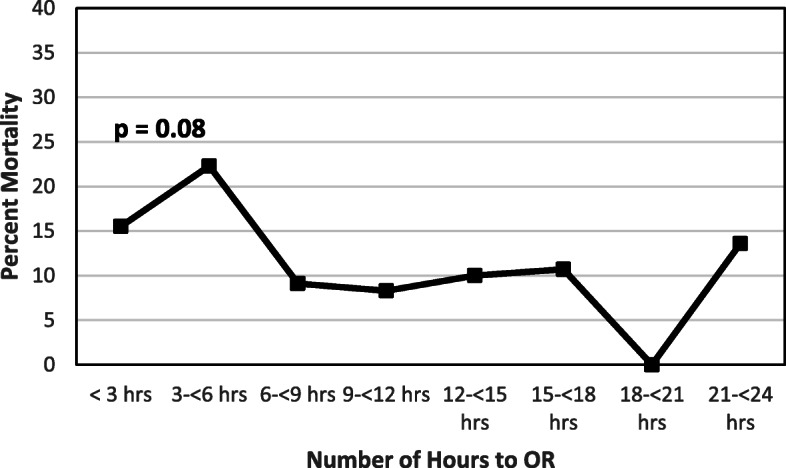
Mortality with Time to Operative Source Control

There was no significant correlation between time to operative source control and mortality (*p* = 0.08, Fig. 3).

Patients were additionally grouped into less than and greater than or equal to 30 mL/kg of fluid resuscitation. There were 203 patients (67%) who received less than 30 mL/kg of fluid, and 98 patients (33%) who received greater than or equal to 30 mL/kg (Table 3). These patient groups were similar in age and gender. Each group had similar presenting heart rates, medians 96 and 101 (*p* = 0.21), however, had significant differences in mean arterial pressure, 88 mmHg vs 82 mmHg (*p* < 0.001), SBP, 124 mmHg vs 113 mmHg (*p* = 0.001) and initial lactate, 1.8 vs 2.3 (*p* = 0.002). These groups scored similar on qSOFA, 1 vs 1 (*p* = 0.06). The patients in the < 30 mL/kg group received significantly less fluid, with median fluid/kg 12.9 vs 46.9 (*p* < 0.001). Despite more resuscitation, patients ≥30 mL/kg more frequently required vasopressors 37% vs 24% (*p* = 0.02).

**Table 3:** < 30 mL/kg vs ≥ 30 mL/kg. Reported as standard deviations unless otherwise stated

	< 30 ml/kg (***n*** = 203)	≥30 ml/kg (***n*** = 98)	***p***-value
	Median (IQR)	Median (IQR)	
**Age**	55 (41–68)	56 (44–71)	0.46
**Male, n (%)**	106 (52%)	46 (47%)	0.39
**HR**	96 (82–112)	101 (85–118)	0.21
**MAP**	88 (75–102)	82 (70–93)	< 0.001
**SBP**	124 (109–140)	113 (102–129)	0.001
**Lactate (*n*** **= 166/81)**	1.8 (1.2–2.6)	2.3 (1.5–4.4)	0.002
**Total Fluids**	12.9 (4.7–20.0)	46.9 (37.8–64.7)	< 0.001
**Preoperative Vasopressor**	49 (24%)	36 (37%)	0.02
**Time to OR (Hours)**	6.1 (3.5–12.2)	4.9 (2.7–13.1)	0.11
**Sofa**	2 (0–7)	3 (1–10)	0.05
**qSOFA**	1 (0–1)	1 (0–2)	0.06
**LOS (d)**	8 (3–19)	9 (3–23)	0.57
**ICU LOS (d)**	2 (0–11)	4 (0–13)	0.22
**Ventilator Days**	1 (0–6)	3 (0–7)	0.08
**Mortality**	23 (11%)	21 (21%)	0.02

Mortality was significantly lower in patients who received less fluid, 11% vs 21% (*p* = 0.02). Median Ventilatory days (1 vs 3), ICU LOS (2 vs 4) and hospital LOS (8 vs 9) were all lower with fewer fluids, however the differences were not statistically significant.

Further analyses compared patients who survived versus non-survivors (Table 4). Survivors had a significantly lower average age 53 vs 65 (*p* < 0.001) and presented with lower SOFA 2.0 vs 10.5, (*p* < 0.001) and qSOFA (1.0 vs 2.0, *p* < 0.001) scores. Survivors presented with better vital signs, including higher MAP (90 mmHg vs 72 mmHg, *p* < 0.001), lower heart rates (96 bpm vs 105 bpm, *p* = 0.02) and a lower respiratory rate (18 vs 22 breaths per minute, *p* = 0.005). Survivors had better baseline physiology and renal function with a lower lactate (1.7 vs 3.2 mmol/L, *p* < 0.001) and creatinine (1.0 vs 2.0 mg/dl, *p* < 0.001). Patients who survived required on average lower volumes of fluid per kilogram (17.8 vs 27.4 mL/kg, *p* = 0.007) and only 21% of survivors compared with 68% of non-survivors required pre-operative vasopressors (*p* < 0.001). Lastly, non-survivors tended to have operative source control quicker than survivors (4.1 h vs 6.2 h, *p* = 0.06).

**Table 4:** Comparison of variables of survivors vs non-survivors, expressed as medians and interquartile range, except otherwise noted

Survivors vs Non-Survivors			
	Survived (*n* = 257)	Died (*n* = 44)	*p*-value
Demographics			
Age (years)	53 (38–67)	65 (58–72)	< 0.001
Vitals			
HR (beats/minute)	96 (83–112)	105 (93–122)	0.02
MAP (mmHg)	90 (76–101)	72 (66–79)	< 0.001
SBP (mmHg)	123 (109–139)	106 (91–116)	< 0.001
RR (breaths/minute)	18 (16–22)	22 (18–29)	0.005
Temp (°F)	98.4 (98.1–99.3)	98.1 (96.6–99.9)	0.24
Laboratory Values			
Bilirubin (mg/dL)	0.9 (0.6–1.4)	1.5 (1.1–2.5)	< 0.001
Creatinine (mg/dL)	1.0 (0.8–1.4)	2.0 (1.4–3.2)	< 0.001
Lactate(mmol/L)	1.7 (1.2–2.6)	3.2 (1.8–6.9)	< 0.001
Platelet (×10^3^/mcL)	240 (175–314)	167 (97–283)	0.001
Scoring Systems			
SOFA	2.0 (0–6)	10.5 (6–14)	< 0.001
qSOFA	1.0 (0–1)	2.0 (1–3)	< 0.001
Interventions			
Pre-op Vasopressor	55 (21%)	30 (68%)	< 0.001
Time to OR (hours)	6.2 (3.3–13.0)	4.1 (3.3–7.6)	0.06
Fluids per Kg (mL/kg)	17.8 (8.6–32.7)	27.4 (14.3–59.6)	0.007

A regression model measuring the unadjusted effect of binary fluid level on mortality on the cohort of 301 patients (Table 5) indicated that patients treated with 30 or more mL/kg were 1.89 times as likely to die than those treated with fluids below 30 mL/kg (95% CI 1.10–3.25, *p* = 0.02). Upon adjustment by covariates (age, MAP, SBP, heart rate, lactate, creatinine and SOFA score), the amount of fluid administered was no longer statistically significant (*p* = 0.59). Significant risk factors for mortality in the adjusted model included increasing age (*p* = 0.001), increasing lactate (*p* < 0.001), and increasing SOFA (*p* < 0.001) levels. The inclusion of lactate in the latter model reduced the patient sample size in the model to 247. In a separate model without lactate (*N* = 299), increasing age and SOFA remained as the only statistically significant risk factors for death.

**Table 5:** Relative risk regression model for mortality for those treated with < 30 or ≥ 30 ml/kg, and adjusted for covariate risk factors

Relative Risks for Risk Factors of Mortality Effect of Fluid Volume 30+ ml/kg vs. < 30 ml/kg				
		**95% CI for RR**		
**Unadjusted Model (*N*** **= 301)**	**RR**	**Lower**	**Upper**	***p*-value**
30+ ml/kg vs. < 30 ml/kg	1.89	1.10	3.25	0.02
		**95% CI for RR**		
**Adjusted Model (*N*** **= 247)**	**RR**	**Lower**	**Upper**	***p*-value**
30+ ml/kg vs. < 30 ml/kg	1.15	0.69	1.92	0.59
Age (years)	1.03	1.01	1.05	0.001
MAP (mmHg)	0.99	0.97	1.02	0.63
SBP (mmHg)	1.00	0.98	1.02	0.77
Heart rate (beats/minute)	1.01	1.00	1.02	0.20
Lactate (mmol/L)	1.09	1.05	1.14	< 0.001
Creatinine (mg/dL	1.00	0.86	1.17	0.95
SOFA	1.13	1.07	1.19	< 0.001

Similar results were found when analyzing the effect of increasing volume by 10 ml/kg intervals on mortality (Table 6). An unadjusted model indicated that mortality risk significantly increased by 14% for every 10 mL/kg increase in fluid volume (RR = 1.14, 95% CI 1.07–1.21, *p* < 0.001). Fluid volume was no longer significant (*p* = 0.78) when adjusted by the covariates listed above, replaced by increasing age (*p* = 0.002), lactate (*p* < 0.001) and SOFA (*p* < 0.001) levels as significant risk factors in a model with lactate and by increasing age and SOFA in a separate model without lactate.

**Table 6:** Relative risk regression model for mortality for those treated with increasing amounts of fluid by 10 mL/kg and adjusted for covariate risk factors

Relative Risks for Risk Factors of Mortality Effect of Fluid Volume in Increasing 10 ml/kg Intervals				
		**95% CI for RR**		
**Unadjusted Model (*N*** **= 301)**	**RR**	**Lower**	**Upper**	***p*-value**
10 ml/kg Increase in Fluid Volume	1.14	1.07	1.21	< 0.001
		**95% CI for RR**		
**Adjusted Model (*N*** **= 247)**	**RR**	**Lower**	**Upper**	***p*-value**
10 ml/kg Increase in Fluid Volume	1.01	0.93	1.10	0.78
Age (years)	1.03	1.01	1.05	0.002
MAP (mmHg)	0.99	0.97	1.02	0.65
SBP (mmHg)	1.00	0.98	1.02	0.74
Heart rate (beats/minute)	1.01	1.00	1.02	0.20
Lactate (mmol/L)	1.10	1.05	1.15	< 0.001
Creatinine (mg/dL	1.02	0.88	1.17	0.83
SOFA	1.12	1.07	1.19	< 0.001

## Discussion

This study represents one of the first studies examining the amount of pre-operative fluid resuscitation in patients who need urgent source control of intra-abdominal infection. We sought to answer the age-old question, of how much resuscitation is needed prior to surgery. Our initial data analysis demonstrates that increasing fluid resuscitation prior to source control was independently associated with a significant increase in mortality (Fig. 2). This study aligns with other reports showing an increase in mortality with increasing fluids [6–8, 12, 13] and others advocating for trials of decreasing fluid resuscitation [14, 15]. This data differed when adjusting for covariates. In the unadjusted model, patients who received greater than 30 ml/kg of volume had and increased mortality by 1.89 times, however when adjusting for covariates (age, MAP, SBP, heart rate, lactate, creatinine and SOFA score, Table 5) increasing fluid resuscitation was no longer associated with mortality in our multivariate model. This change in significance is likely the result of sicker and more physiologically deranged patients receiving and requiring more fluids for adequate pre-operative resuscitation.

With the thought that ‘sicker’ patients require more fluid resuscitation, we sought to compare how ‘sick’ each group was. In addition to the multivariate model, we separated our sample size into two groups, based on the Surviving Sepsis Campaign resuscitation guidelines of those who received < 30 mL/Kg or ≥ 30 mL/kg. Both groups had similar demographics with respect to male/female (52% vs 47%) and similar age (55 vs 56). These groups were similar in qSOFA scores, however the presenting physiology differed. The patients who received more fluids had significantly greater severity of illness on admission, with lower MAP (82 vs 88), lower SBP (113 vs 124), and higher lactate (2.3 vs 1.8). Patients with worse physiology received more preoperative fluid resuscitation and required more vasopressors prior to the operating room. Interestingly, more fluids did not translate into delay to operative source control, as these patients had similar times to operation (4.9 vs 6.1 h). When examining outcomes, patients who received more fluids tended to have increased ventilator days, ICU and hospital lengths of stay, though none of these comparisons were statistically significant. Patients who received more fluids had nearly double the mortality rate, 21% vs 11% (*p* = 0.02).

Comparing survivors vs non-survivors, non-survivors were older and had greater severity of illness demonstrated by worse presenting physiology. Non-survivors were more tachycardic (105 vs 96 beats/min), tachypneic (22 vs 18 breaths/min), and hypotensive (SPB 106 vs 123 mmHg) compared to survivors. Physiology was deranged as well, with non-survivors on average having higher lactate, 3.2 vs 1.7, and baseline organ dysfunction, Cr 2.0 vs 1.0 mg/dl, bilirubin 1.5 vs 0.9 mg/dl. Severity of illness scores were worse in non-survivors, with presenting SOFA scores of 10.5 vs 2.0, and qSOFA scores of 2.0 vs 1.0. These findings align with the current knowledge that physiologically deranged patients, aka ‘sicker’, do worse [2, 9, 10, 16, 17]. Additionally, sicker patients often require more fluids, as was the case in our series.

The relative risk regression model, without adjusting for lactate we demonstrated significant increases in mortality with greater fluid administration (Tables 5 and 6). However, when the model was adjusted for lactate, the only significant increases in mortality were increasing age, increasing lactate, and higher presenting SOFA scores (Tables 5 and 6). Volume of resuscitation did not have a significant impact on mortality. This differs some recent reports showing high fluid administration and positive fluid balances increasing mortality [6–8]. With respect to factors associated with worsening outcomes, out study aligns with current reports and scoring systems that show increasing age, worse physiology and organ failure scores are associated with increased mortality [2, 9, 10, 16, 17].

There were several limitations of this study. First of the 301 patients that met inclusion criteria, all patients were resuscitated either prior to transport to our facility, at our University Hospital, or by a combination of resuscitation at both locations. One limitation of our study, due to the retrospective nature of our study, was that there were no controls on the types of crystalloid fluid (normal saline, lactated ringers, or Plasma-Lyte) administration used for resuscitation. Additionally, patients had varying places of resuscitation, requiring resuscitation in outside emergency departments, outside hospitals, our university emergency room, or our university critical care resuscitation unit which may have impacted the total amount of fluid administration. Differences in outcome per type of fluid administered with relation to balanced vs normal saline were not recorded and may have impacted results as seen in the Balanced Crystalloids versus Saline in Critically Ill and Noncritically Ill adults (SMART) and Saline against Lactated Ringer’s or Plasma-Lyte in the Emergency Department (SALT-ED) trials [18–20].

Additionally, the timing of administration, type of vasopressor used, and total amount of vasopressors were not captured with our database. Thus vasopressor use was not uniform throughout the study time period and varied by each treating physician which may have an impact on results [1, 21]. Thirdly, there was no uniform protocol for operative intervention in these patients, and need for operative source control was determined by the attending surgeon which may have impacted times to operative intervention. Lastly, we did not collect time to antibiotic administration or presence of antibiotic administration, which may have contributed mortality in these septic patients [1, 16].

The final limitation in our study relates to how ‘sick’ our patients were. Sepsis 3 defines sepsis as qSOFA > 2 with septic shock as qSOFA > 2, Lactate > 2 and/or vasopressor use [5]. A large proportion of our patients did not meet these criteria for sepsis or septic shock and thus may have done with will any volume of pre-operative fluid resuscitation. Future studies will be needed to look specifically at the population of patients who are septic or are in septic shock.

## Conclusion

In our cohort of patients, using an adjusted regression model, we did not find a significant association between the amount of pre-operative fluid resuscitation and mortality but observed significant increases in mortality with increased age, lactate levels, and SOFA scores. The amount of pre-operative volume resuscitation was not associated with differences in time to operative source control, ventilator days, ICU or total hospital LOS. Patients who died had deranged physiology and evidence of organ dysfunction on admission. In our series the amount of preoperative fluid resuscitation was not associated with mortality, showing that the total amount of preoperative fluid resuscitation does not impact mortality. This one of the first studies of its kind examining pre-operative fluid resuscitation, but further studies are needed to answer the question of what is the optimal amount of preoperative fluid resuscitation in the septic emergency surgery patient.

## Data Availability

The datasets analyzed during the current study are not available publicly, as they are part of a prospectively collected database. The de-identified dataset is available on request from the corresponding author.

## Abbreviations

EGS: Emergency General Surgery (EGS)
ICU: Intensive Care Unit
SOFA: Sequential Organ Failure Assessment Score
qSOFA: Quick Sequential Organ Failure Assessment Score
IQR: Interquartile range
BMI: Body mass index
MAP: Mean arterial pressure
SBP: Systolic blood pressure
FiO2: Fractional inspired oxygen
PaO2: Partial pressure of oxygen
LOS: Length of stay
